# Egg By-Products as a Tool to Remove Direct Blue 78 Dye from Wastewater: Kinetic, Equilibrium Modeling, Thermodynamics and Desorption Properties

**DOI:** 10.3390/ma13061262

**Published:** 2020-03-11

**Authors:** Ainoa Murcia-Salvador, José A. Pellicer, María Isabel Rodríguez-López, Vicente Manuel Gómez-López, Estrella Núñez-Delicado, José A. Gabaldón

**Affiliations:** Dpto. de Ciencias de la Salud., Universidad Católica San Antonio de Murcia (UCAM), Avenida de los Jerónimos s/n, 30107 Murcia, Spain; amurcia6@alu.ucam.edu (A.M.-S.); japellicer@ucam.edu (J.A.P.); mirodriguez@ucam.edu (M.I.R.-L.); vmgomez@ucam.edu (V.M.G.-L.); enunez@ucam.edu (E.N.-D.)

**Keywords:** adsorption, eggshell, Direct Blue 78, kinetics, isotherms, pulsed light

## Abstract

Eggshell, a waste material from food manufacturing, can be used as a potential ecofriendly adsorbent for the elimination of textile dyes from water solutions. The adsorption process was evaluated varying factors such as initial dye load, contact time, pH, quantity of adsorbent, and temperature. The initial dye load (Direct Blue 78) was in the range of 25–300 mg/L. The kinetics of adsorption were analyzed using different models, such as pseudo-first-order, pseudo-second-order, and intraparticle diffusion model. Also, the experimental data at equilibrium were studied using Freundlich, Langmuir, and Temkin isotherms. The kinetics followed pseudo-second-order, then pseudo-first-order, and finally the model of intraparticle diffusion. The results obtained for data at equilibrium follow the order: Freundlich > Langmuir > Temkin. The adsorption equilibrium showed a maximum capacity of adsorption (q_max_) of 13 mg/g at pH 5, and using 0.5 g of eggshell. Dye adsorption was enhanced with increasing temperatures. The thermodynamic study revealed the spontaneity and endothermic nature of the adsorption process. The desorption study shows that the eggshell could be reused in different adsorption/desorption cycles. A novel advanced oxidation process could degrade more than 95% of the dye. The results show that eggshell is a waste material useful to remove hazardous dyes from wastewater, which may alleviate the environmental impact of dyeing industries.

## 1. Introduction

Wastewater polluted with large amounts of dyes is usually dumped into aqueous effluents from the food and plastic, leather, paper, printing, and textile industries [1]. Dyes have been widely used in many industries for coloration purposes thanks to their good features, such as easy application, low production cost, bright colour, and water-fastness [2]. Nowadays, more than 100,000 commercial dyes exist with over 7 × 10^5^ tons of dyes produced per year, and approximately 10–15% are discharged from textile industries [3]. Dyes are organic compounds that are classified according to the chemical composition and type of application. Commercial dyes are categorized into three classes: cationic, anionic, and nonionic, according to the charge after its dissolution in water. Azo compounds are the most typical dyes used for industrial purposes [4,5,6].

Dyes are non-biodegradable, stable, oxidizing agents and are highly toxic and mutagenic to aquatic life and humans [7]. Their discharge may produce severe hazard to aquatic living organisms, affecting different processes of aquatic vegetation, decreasing the oxygen levels in water and resulting in the choking of aquatic fauna and flora [8]. Thus, it is essential to reduce and remove organics pollutants from wastewater before discharging them [9]. Various methods have been applied for the removal of harmful contaminants from water and wastewater, involving adsorption on activated carbons, reverse osmosis, chemical oxidation, membrane filtration, bacterial action, coagulation and flocculation, activated sludge, ozonation, precipitation, electro-dialysis, ion exchange, and electrochemical techniques [10,11]. However, many of these methods are expensive and exhibit other drawbacks such as poor effectiveness and formation of sludge excess, and are thus unsuitable to be applied by small-scale industries [1]. Between them, adsorption is considered the most feasible method and has become one of the effective and easiest physico-chemical treatment procedures for the decolourization of textile wastewater. It offers several advantages such as low initial costs, high efficiency, producing nontoxic intermediates or by-products, high removal capability, versatility, easy handling, fast adsorption rate, and facile separation [2,12].

The adsorption based on activated carbon is widely employed to remove dyes, however, it still remains an expensive process owing to its high initial cost and the need for a regeneration system of the adsorbent that make it less economically viable [8]. Recently, more effective and cheaper adsorbents based on by-products from poultry waste, agricultural waste, and other natural waste have been developed as an alternative to conventional wastewater treatment processes [13].

In the last decades, researchers have been paying attention to the adsorption properties of agricultural wastes to develop new biosorbents in accordance with circular economy principles. Some alternatives include the orange peel, chitosan, eggshell, grape pomace, coffee residues, rice straw, olive stones, banana peel, artichoke agrowaste, sugarcane bagasse, and bamboo shell, among others [14,15,16,17,18,19,20,21,22,23,24]. This waste, which is eco-friendly, economic renewable, and available in abundance, is a candidate for the treatment of polluted water and wastewater [11]. Among the agricultural poultry by-products, the waste generated can be a promising biosorbent that has slightly increased in recent years. Moreover, the use of by-products from the agro-food sectors in the adsorption process helps to reduce the waste, as well as the low cost biomasses acquisition [17]. Waste materials obtained from different sources have been used as potential adsorbents for the removal of inorganic and organic pollutants. Eggshell and eggshell membranes are waste materials produced in large amounts in the poultry and farms industries as well as restaurants, bakeries, or homes [25].

Approximately 10% of the total mass of hen egg corresponds to eggshell by-product, with an average weigh of 60 g, and it is usually discarded in landfills without any pretreatment. In addition, this waste is commonly reused as soil conditioner, fertilizer, or additive for animal feed. The eggshell consists of three layers: the cuticle on the outer surface (mucin protein), the calcified eggshell (calcite or calcium carbonate crystals), and the eggshell membrane (protein fibers). Numerous pore channels are distributed on the surface of eggshell (between 7000 and 17,000 pores), thus eliciting water transpiration and gaseous exchanges. The porous nature of eggshell makes it a desirable material to be used as an adsorbent [11,13].

Retention of contaminants by adsorbents is not 100% efficient; therefore, additional methods are required to avoid non-retained dyes being disposed of into the environment. The degradation of dyes by an advanced oxidation process (AOP), using pulsed light technology as a photolyzer of hydrogen peroxide, is a novel, fast, and efficient version of AOPs that uses mercury-free lamps to generate energy-dense light [26].

Thus, the main objective of this paper was to evaluate the potential use of eggshell to remove Direct Blue 78 (DB78) dye in aqueous solution by adsorption, and to determine the effect of different parameters such as contact time, initial dye load, pH, and adsorbent concentration. The isotherms of adsorption, as well as the kinetics of dye adsorption on the eggshell, were evaluated by fitting the experimental data to different kinetics and isotherms models. Further, the efficiency of degradation of DB78 by a novel AOP in order to minimize dye discharge to the environment was measured.

## 2. Materials and Methods

### 2.1. Materials

Hen eggshells were purchased from a local organic farm. Sodium hydroxide (NaOH), acetic acid (C_2_H_4_O_2_), sodium phosphate monobasic monohydrate (H_4_NaO_5_P), hydrogen peroxide (H_2_O_2_), and hydrochloric acid (HCl) were obtained from Sigma-Aldrich (Madrid, Spain) and boric acid (H_3_BO_3_) was purchased from Scharlau (Madrid, Spain). All of these chemicals were used to prepare dye solutions at different pH values. Colorprint (Valencia, Spain) kindly provided Direct Blue 78 dye. Deionized water was utilized to prepare all aqueous solutions throughout the experiments.

### 2.2. Eggshell Conditioning

Eggshells with their membrane were washed under tap water, dried at room temperature, and stored at −35 °C in order to prevent the spoilage of the eggshell samples before using them. Before using them, eggshells were ground in a sturdy vessel in which the material was pounded with a pestle and sieved to obtain the required particle size (1–1.5 cm^2^). Thereafter, pieces of eggshell were used as the adsorbent in the adsorption studies.

The major constituents of the eggshell are carbonates, sulphates and phosphates of calcium and magnesium, and organic matter. Traces of Na, K, Mn, Fe, Cu, and Sr metals are also present in the eggshell [27]. The density of the eggshell is about 2.53 g/cm^3^, which is significantly larger than that of eggshell membrane (1.358 g/cm^3^). The major constituents of the eggshell are calcium carbonate (94%), organic matter (4%), calcium phosphate (1%), and magnesium carbonate (1%). The eggshell membrane possesses nearly 60% protein (collagen (35%), glucosamine (10%), chondroitin (9%), and hyaluronic acid (5%)), along with other inorganic components like Ca, Mg, Si, Zn, and so on in smaller quantities [28]. The membrane surface bears positively charged sites produced by basic side chains of amino acids. It has a very high surface area with special functional groups such as hydroxyl (−OH), thiol (−SH), carboxyl (−COOH), amino (−NH_2_), amide (−CONH_2_), and so on, which strongly interact with some chemical species present in the albumen.

### 2.3. Dye Solution Preparation

DB78 is an azo dye (CAS 2503-73-3) whose molecular weight is 1055.91 g/mol and whose formula is C_42_H_25_N_7_Na_4_O_13_S_4_. Aqueous solutions with different concentrations of dye (25, 50, 100, 150, 200, 250, and 300 mg/L) were prepared from a 1 g/L stock solution, and used to determine the capacity of adsorption of the eggshell.

### 2.4. Analyses and Data Evaluation

The concentration of DB78 was measured at the wavelength of 612 nm, which is the maximum absorbance of the dye [15]. A spectrophotometer was used to determine the absorbance before and after the treatment (UV-1603, Shimadzu, Kyoto, Japan).

### 2.5. Adsorption Experiments

The adsorption experiments were conducted at different dye concentrations, ranging from 25 to 300 mg/L. The kinetic adsorption studies were performed at different doses of adsorbent, pH, and dye concentrations. The effect of contact time on the adsorption capacity of eggshell was conducted while varying dye concentrations from 25 to 300 mg/L. Furthermore, the effect of adsorbent dosage was studied to optimize the adsorption process. pH was carefully adjusted between 3 and 11 to determine the optimum pH to ensure the maximum DB78 removal. Apart from that, the effect of temperature on the adsorption of DB78 by eggshell was investigated at three different temperatures (29, 55, and 75 °C).

A typical experiment was conducted by adding the eggshell to 40 mL of different dye concentrations of the DB78 solution. The mixture was stirred for a predetermined period of time at a constant speed of 500 rpm. The residual dye concentration in the solution was measured at time intervals (20, 40, 60, 80, 100, 120, and 140 min) until the equilibrium conditions were attained. At the end of each interval, the samples were centrifuged at 3000 rpm for 5 min to separate the solid phase, completely removing impurities that may later affect the measure.

The amount of dye adsorbed on eggshell (*q_t_*) at time (*t*), in mg/g, was determined by Equation (1), as follows [29]:

(1) $$q_{t}=\frac{V\;\left(C_{0}-C_{e}\right)}{m}$$

where *V* is the volume of dye solution (L); *C*_0_ and *C_e_* are the initial and equilibrium concentrations of dye in liquid phase (mg/L), respectively; and *m* is the mass of eggshell (g). All the experiments were carried out in triplicate. Three adsorption kinetics models, three isotherms, and the thermodynamic study were evaluated to elucidate the mechanism of dye adsorption.

### 2.6. Adsorption Kinetics

In order to analyze the mechanism of dye adsorption onto eggshell, and to predict the rate at which a solute (dye) was removed from aqueous solution, three kinetic models could be employed: pseudo-first-order kinetic model [30], pseudo-second-order kinetic model [31], and intraparticle diffusion model [32].

The Lagergren’s equation for pseudo-first-order kinetics is given by the following Equation (2) [30]:

(2) $$\log\left(q_{e}-q_{t}\right)=\log q_{e}-\frac{k_{1}}{2.303}t$$

where *q_e_* and *q_t_* are the adsorption capacity at equilibrium and at time (*t*) (mg/g), respectively, and *k*_1_ (min^−1^) is the rate constant of this model. From the linear plot of log (*q_e_* − *q_t_*) versus *t*, the rate constant (*k*_1_) can be obtained by the slope.

The linear form of the Ho and McKay rate equation for pseudo-second-order kinetics is expressed as Equation (3), as follows:

(3) $$\frac{t}{q_{t}}=\frac{1}{k_{2}q_{e}{}^{2}}+\frac{1}{q_{e}}t$$

where *q_e_* and *q_t_* are the adsorption capacity (mg/g) at equilibrium and at time (*t*), respectively, and *k*_2_ (g/mg min) is the rate constant of this model and can be obtained from the intercept and slope of plot *t/q_t_* versus *t* [31].

In the adsorption experiments, it is mandatory to fit the experimental data to the intraparticle diffusion model in order to analyze in depth the adsorption behavior of DB78 on eggshell, and to know the rate-determining step in the liquid adsorption systems. In the adsorption of pollutants onto adsorbents, different stages are differentiated; in the first one, there is a transport of the dye from the solution to the adsorbent surface, followed by the diffusion into the adsorbent, which is usually a slow process [32]. In the diffusion model proposed by Weber and Morris [33], the rate can be expressed in terms of the square root time and can be determined as Equation (4), as follows:

(4) $$q_{t}=k_{i}\sqrt{t}+C$$

where *q_t_* is the adsorption capacity at any time *t* (mg/g); and *k_i_* is the rate constant of this model (mg/g min^1/2^) and its values can be calculated from the slopes of plots *q_t_* versus *t*^1/2^, where *t* is the time (min) and *C* is the intercept (mg/g).

### 2.7. Isotherm Analysis

The interaction between dyes and the adsorbent materials is described using different theoretical models, known as adsorption isotherms. These isotherms are essential in the optimization of the adsorption process [34,35]. Equilibrium isotherm equations were used to describe experimental sorption data [36] and the parameters of equilibrium isotherms provide useful information on adsorption mechanisms, affinity of the adsorbent, and surface properties [20]. Different isotherms were used to analyze the adsorption equilibrium in this study: The Freundlich, Langmuir and Temkin models.

The Freundlich isotherm model suggests heterogeneity in the adsorption sites and takes into account that the adsorption occurs at sites with different energy of adsorption. This isotherm is obtained from the linear form of the Freundlich expression Equation (5) [36]:

(5) $$ln\;q_{e}=\ln K_{F}+\frac{1}{n_{F}}\ln C_{e}$$

where *C_e_* (mg/L) and *q_e_* (mg/g) are the liquid phase concentration and solid phase concentration of dye at equilibrium, respectively; 1/*n_F_* is the heterogeneity factor; and *K_F_* is the Freundlich constant (L/g) related to the bonding energy. 1/*n_F_* and *K_F_* values were calculated from the slope and intercept of plots ln*q_e_* versus ln*C_e_*, respectively. The values of 1/*n_F_* indicate the type of adsorption process: favorable (0 < 1/*n_F_* < 1), unfavorable (1/*n_F_* >1), or irreversible (1/*n_F_* = 0) [37].

The Langmuir isotherm model assumes that the adsorption process happens at specific homogeneous sites on the adsorbent. This model is probably the most employed adsorption isotherm and is used successfully for the adsorption of contaminants from water solutions. The linearized form of Langmuir model can be given as follows Equation (6) [33,38]:

(6) $$\frac{C_{e}}{q_{e}}=\frac{1}{K_{L}}+\frac{a_{L}}{K_{L}}C_{e}$$

where *C_e_* is the dye concentration at equilibrium in solution (mg/L), *q_e_* is the adsorption capacity (mg/g) at equilibrium time, and *K_L_* (L/g) and *a_L_* (L/mg) are the Langmuir isotherm constants. The constants *K_L_* and *a_L_* can be calculated from the intercept (1/*K_L_*) and the slope (*a_L_*/*K_L_*) of the linear plot between *C_e_*/*q_e_*, and *C_e_*. *q_max_* is the maximum adsorption capacity of adsorbent (mg/g) and is determined by *K_L_*/*a_L_*.

The fundamental characteristic of Langmuir isotherm is the separation factor, which is a dimensionless constant (*R_L_*), and is given as follows Equation (7) [39]:

(7) $$RL=\frac{1}{1+a_{L}C_{o}}$$

where *C_o_* is the initial dye concentration (mg/L) and *a_L_* is the Langmuir constant related to the energy of adsorption (L/mg). The calculated *R_L_* values indicate the type of adsorption: unfavourable process (*R_L_* > 1), linear (*R_L_* = 1), favourable (*R_L_* between 0 and 1), or irreversible (*R_L_* = 0) [40].

The Temkin formula determines that the decrease of adsorption heat with coverage is linear because of some adsorbate/adsorbent interactions. The adsorption is characterized by uniform distribution of bond energies, up to a maximum bond energy [41]. The linear form of Temkin isotherm’s equation can be expressed as Equation (8), as follows:

(8) $$q_{e}=\frac{RT}{b_{T}}\ln a_{T}+\frac{RT}{b_{T}}\ln C_{e}$$

where *T* is the absolute temperature (K); *R* is the universal gas constant (8.314 J/mol K); *a_T_* is the constant of Temkin isotherm (L/g); *b_T_* is the Temkin constant related to the heat of adsorption (J/mol); and *q_e_* and *C_e_* are the equilibrium concentration of DB78 on eggshell (mg/g) and in the solution (mg/L), respectively. The Temkin constants *a_T_* and *b_T_* values can be calculated from the slope and intercept of straight line plot of *q_e_* versus ln*C_e_.*

### 2.8. Thermodynamic Study

The thermodynamic analysis is needed to conclude whether the adsorption process of the dye onto eggshell is exothermic or endothermic. In order to gain further insight related to these experiments, it is essential to calculate the value of Δ*H*° (standard enthalpy change), Δ*S*° (standard entropy change), and Δ*G*° (Gibbs standard free energy change). The values for the different thermodynamic parameters can be calculated using the thermodynamic equilibrium coefficient obtained at different concentrations and temperatures. The Δ*G*° value is the fundamental parameter to elucidate the spontaneity of the adsorption process, and reactions occur spontaneously when the value of Δ*G*° is negative [34].

Considering the exchange adsorption, the equation employed to calculate the *K°* value at different temperatures was as follows:

(9) $$K{}^{\circ}=K_{p}\times M_{adsorbate}\times 55.5$$

where *K_p_* is the equilibrium constant at time *t* (L/g), *M_adsorbate_* is the molecular weight of DB78 (g/mol), and 55.5 (mol/L) is the mole concentration of water [42].

Using the *K*° values obtained from the previous equation, the Gibbs free energy was determined using the following equation:

(10) ΔG° = −RTlnK°

To confirm the results for the Gibbs free energy, the Van’t Hoff [12] equation was graphed (ln*K*° vs. 1/*T*). Plotting ln*K*° versus 1/*T* gives a straight line with slope and intercept equal to −Δ*H*°/*RT* and Δ*S*°/*R*, respectively, where *R* is the universal gas constant (8.314 J/mol K) and *T* is the absolute temperature in Kelvin (K). Using this representation, the Gibbs free energy was calculated again using the following equation:

(11) ΔG° = ΔH°−TΔS°

### 2.9. Desorption and Regeneration of the Adsorbent

Desorption studies help to recover the adsorbate and adsorbent. Thus, the regeneration of the adsorbent may be important to reduce cost processes and to recover the pollutant extracted from the solution [37]. The viability of desorption and reuse of eggshell was evaluated using 0.5 M NaOH solution. First, 0.5 g of eggshell was stirred with 40 mL of dye (100 mg/L, pH 5), at 25 °C, 100 min of contact time, and 500 rpm for the adsorption phase. The adsorbent was dried and added into 40 mL of NaOH solution for desorption at a constant speed of 500 rpm for 100 min at 25 °C. The dye concentration in the solution was measured at 100 min, after the centrifugation of the samples at 3000 rpm for 5 min to separate the solid phase.

### 2.10. Degradation of DB78 by an Advanced Oxidation Process

The adsorption experiment was carried out using different dye concentrations. After 140 min of contact time and 300 mg/L of dye, the remaining dye in the solution was 144 mg/L. The experiments were performed in triplicate, which means that 144 mg/L was the average of the three repetitions. Then, 18 mL of the unadsorbed dye solution was mixed with 2 mL of a H_2_O_2_ solution that rendered a final H_2_O_2_ concentration of 840 mg/L. This rendered an H_2_O_2_/DB78 ratio of 200 on molar basis, which is enough to avoid making H_2_O_2_ the limiting reagent of the photochemical reaction. The mixture was placed in a pulsed light system (XeMaticA-Basic-1L, Steribeam, Germany) operated at 2.5 kV that supplied 2.14 J/cm^2^ per pulse of a broad-spectrum light that included UV light, the spectrum of which has been previously reported [43]. Treatments were prolonged using multiple pulses up to 120 and carried out in duplicate. Samples were withdrawn every five pulses to measure absorbance. Data were normalized and adjusted to pseudo-first order kinetics (Equation (12)) to calculate the degradation rate.

(12) $$\ln\frac{C}{C_{0}}\;=\;-kF$$

where *C* is the concentration at fluence *F* (J/cm^2^), *C*_0_ is the initial concentration, and *k* is the pseudo-first order rate constant (cm^2^/J). Data were processed using Excel 2010 (Microsoft, Redmond, WA, USA).

## 3. Results and Discussion

### 3.1. Effect of Eggshell Dosage 

**Figure 1:**
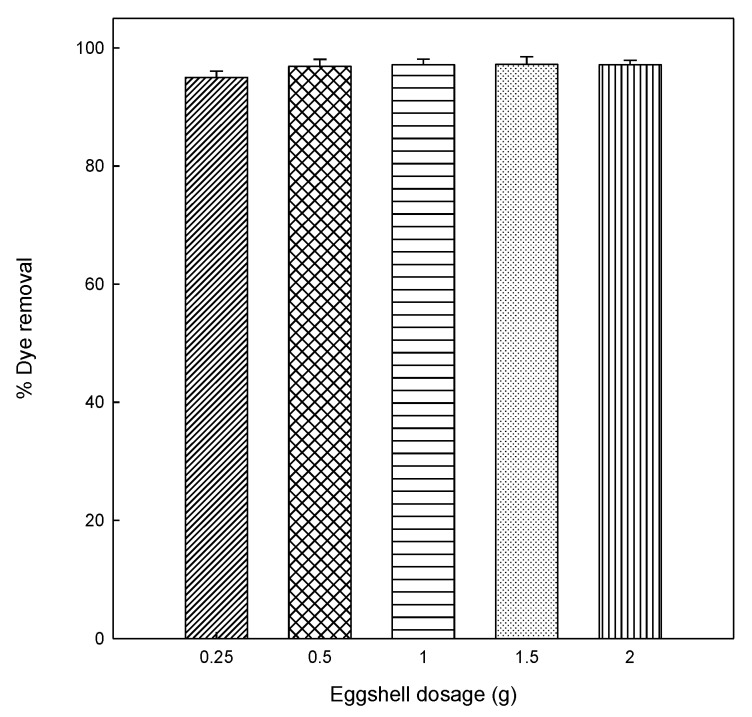
Effect of eggshell amount on the adsorption of Direct Blue 78 (DB78).

Different amounts of eggshell (0.25, 0.5, 1.0, 1.5, and 2.0 g) were added into DB78 solutions to analyze the effect of adsorbent dosage on adsorption. The effect was investigated at an initial concentration of 25 mg/L, pH 5, 120 min of contact time, constant stir (500 rpm), and room temperature (25 °C). The results obtained from this study are shown in the plot of dye removal (%) versus adsorbent dosage (g), as can be seen from Figure 1.

The increase of the adsorbent led to a slight increase for dye removed until a maximum adsorption. This reveals that the removal efficiency of dye increased with increasing amounts of eggshell. At a dosage of 0.5 g, the removal of dye was similar to higher doses (1–2 g), therefore, in order to optimize the adsorption process, and so to use the minimum amount of adsorbent, 0.5 g of eggshell was used in order to study the kinetic and equilibrium experiments.

### 3.2. Effect of Initial pH Solution

**Figure 2:**
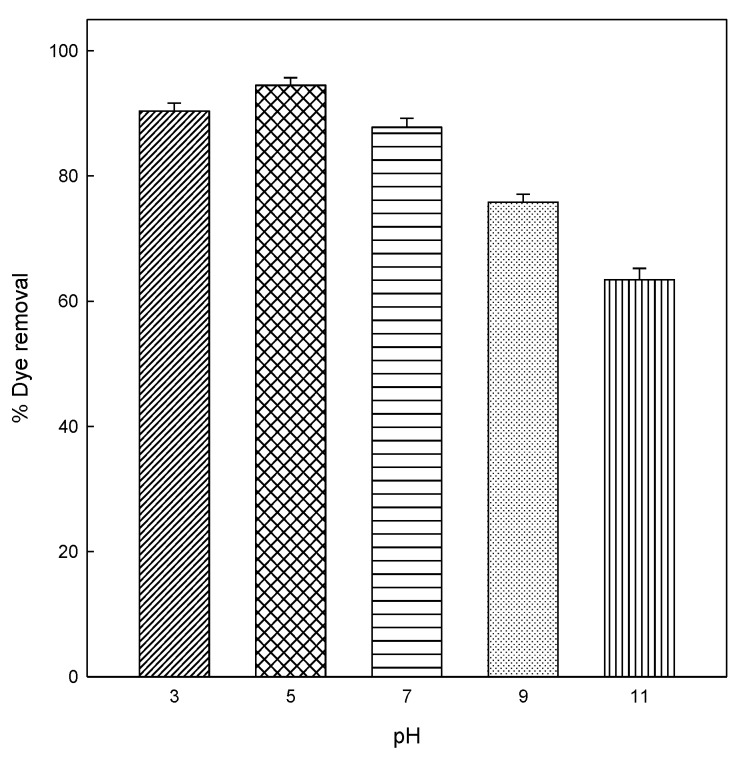
Effect of pH on the adsorption of DB78 by eggshell at different pH values.

The pH of dye solution is considered an essential factor in any adsorption process that affects the adsorption capacity and the adsorption mechanism of the eggshell. With the objective to investigate the effect of the initial dye pH in the adsorption of DB78 dye on the eggshell surface, the equilibrium was studied at different pH values. The effect of pH in the adsorption of DB78 dye was evaluated in the range of pH from 3 to 11. The pH was adjusted by adding NaOH or HCl 0.1 M. The pH experiments were conducted using 2 g eggshell, at a concentration of 25 mg/L, 90 min of contact time, constant stir (500 rpm), and room temperature (25 °C), and the results are shown in Figure 2. The result indicates that the adsorption capacity and removal efficiency of eggshell depend on the pH.

As shown in Figure 2, the removal efficiency of dye increased at acidic pHs (3 and 5). From this pH, the adsorption capability of the eggshell decrease from 94.5% to 63.4% at pH 11. Figure 2 shows that the optimum pH required to obtain the maximum adsorption of DB78 onto eggshell is 5. The isoelectric point for the eggshell is 5.5 [44], which is close to our optimum pH (5.0). At this pH, the adsorbent is positively charged and interacts effectively with the negative charges in DB78. This trend was similar in the removal of Direct Red 80 and Acid Blue 25 dyes from aqueous solutions on eggshell membrane, in which the adsorption capacities of both anionic dyes onto eggshell membrane increased with acidic pHs [9] and, in another study, Chojnacka carried out the biosorption of Cr (III) ions from aqueous solutions by eggshells at pH 5 [45].

The principal component of the shell is CaCO_3_ in the form of the mineral calcite. In water, the carbonate species derived from calcite are H_2_CO_3_, HCO_3_^−^, and CO_3_^2−^, and a sufficient amount of carbonates is solubilized from the shells to buffer the mixtures to a low alkaline pH (from 7.5 to 8) after reaching the equilibrium. Therefore, at various pH, the electrostatic attraction as well as the structure of dye molecules and the eggshell could play very important roles in dye adsorption on this adsorbent. At pH 5, a significantly high electrostatic attraction exists between the positively charged surface of the adsorbent, owing to the ionization of functional groups of adsorbent and negatively charged dye. As the pH of the system increases, the number of negatively charged sites is increased. A negatively charged site on the adsorbent does not favor the adsorption of this dye owing to the electrostatic repulsion [46,47].

### 3.3. Effect of Contact Time

**Figure 3:**
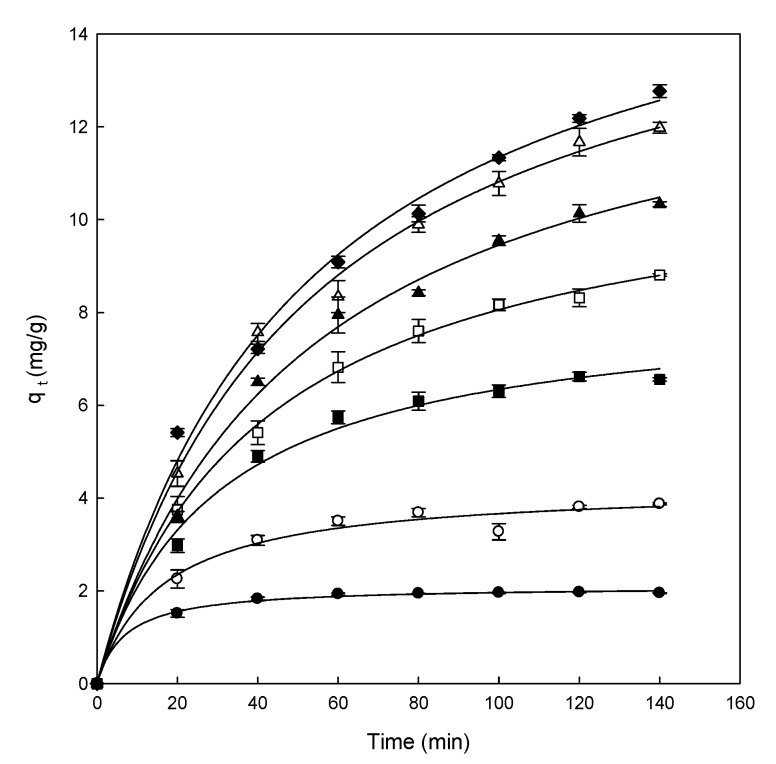
Effect of contact time between eggshell at different concentrations of Direct Blue 78 of 25 mg/L (●), 50 mg/L (○), 100 mg/L (■), 150 mg/L (□), 200 mg/L (▲), 250 mg/L (Δ), and 300 mg/L (♦).

The following step in the adsorption experiments is to elucidate the effect of contact time using different concentrations of dye (from 25 to 300 mg/L). All the experiments were carried out at pH 5.0, constant stir (500 rpm), and room temperature (25 °C), with a fixed amount of adsorbent (0.5 g), and the results obtained are presented in Figure 3.

As can be observed in Figure 3, the adsorption capacity increased in each concentration until the equilibrium was achieved. In the equilibrium, the amount of adsorbed dye inside the adsorbent and the amount of dye desorbed were in a dynamic equilibrium. The time required for the adsorption of the dye onto the adsorbent to attain the equilibrium state is called the equilibrium time and the amount of dye removed by the adsorbent at that time indicates the maximum adsorption capacity of the adsorbent under these conditions [48].

Different adsorption stages can be differentiated in the range of concentrations analyzed (25–300 mg/L). At a low concentration (25 mg/L), the equilibrium was reached after 40 min of contact. The adsorption was fast at the concentration of 100 mg/L, however, it was slower than at low concentrations, reaching the equilibrium after 80 min of contact time. At high concentrations of dye (>100 mg/L), the curves did not present the asymptotic form. Hence, the equilibrium time increased with increasing concentrations of DB78 (from 150 to 300 mg/L).

### 3.4. Adsorption Kinetics

**Figure 4:**
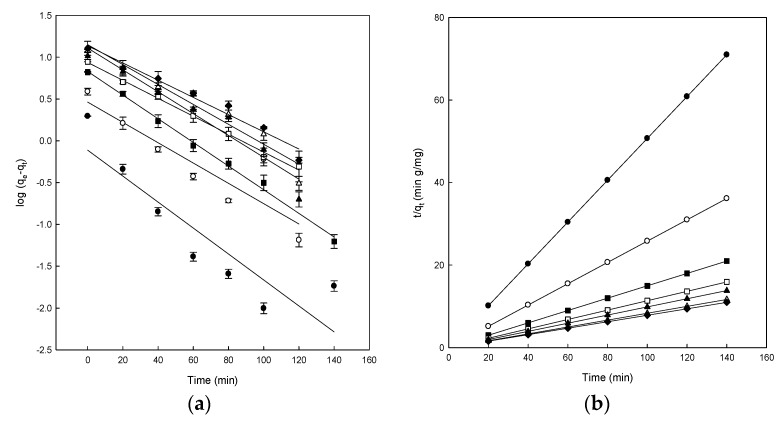

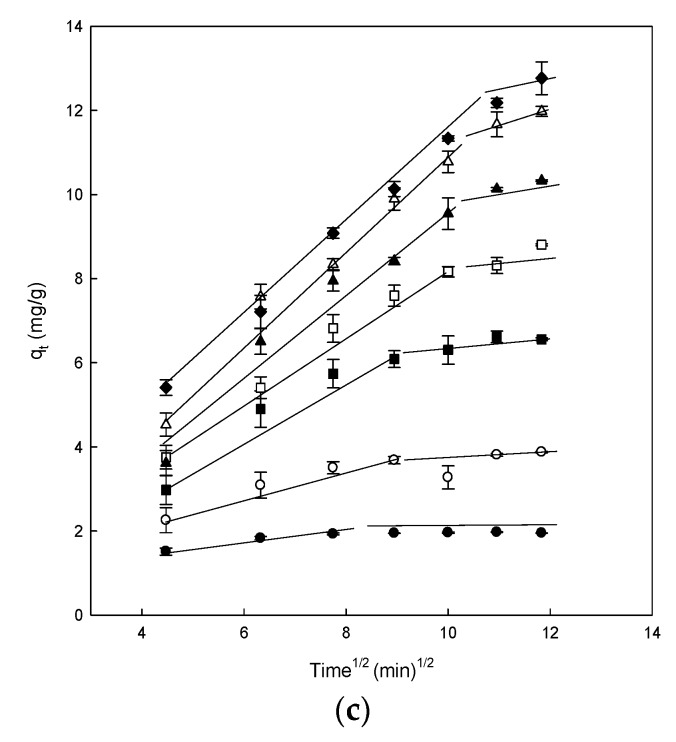
(**a**) Pseudo-first-order model plots, (**b**) pseudo-second-order model plots, and (**c**) intraparticle diffusion model plots for the Direct Blue 78 adsorption onto eggshell at different concentrations of dye of 25 mg/L (●), 50 mg/L (○), 100 mg/L (■), 150 mg/L (□), 200 mg/L (▲), 250 mg/L (Δ), and 300 mg/L (♦).

The adsorption surface, mass transfer, or intraparticle diffusion are different mechanisms involved in the adsorption process and, to study them, three kinetic models were employed to test the experimental data obtained in the adsorption of DB78 on eggshell. The determination coefficient values (R^2^) are essential to decide the best adjustment to the experimental data to the different models tested. The results for the adjustment to the pseudo-first-order, pseudo-second-order, and the intraparticle diffusion kinetic models are shown in Figure 4, and the main parameters for each model are presented in Table 1.

The linearity of the Lagergren model (log(*q_e_* − *q_t_*) versus *t*) was graphed for 140 min of contact with the eggshell (Figure 4a). The *R*^2^ values ranged from 0.800 to 0.995 (Table 1). Calculated values of *q_e_* were compared with the experimental data, and although some *R*^2^ values were relatively high, the *q_e_* values calculated were not suitable. The obtained *R*^2^ and *q_ecal_* values indicated that the adsorption of dye onto eggshell did not follow the pseudo-first-order kinetics, even though some values were relatively high; consequently, this equation cannot be used to analyze the experimental results. Because of the obtained results, it was appropriate to fit the experimental data to the pseudo-second-order model. Figure 4b shows the results obtained after applying the Ho and McKay model. The plot of *t*/*q_t_* versus *t* produced straight lines for the entire measurement range.

The theorical *q_e_* values were identical to the experimental *q_e_* values obtained using the pseudo-second-order, as compared with those of the pseudo-first-order kinetic, indicating that DB78 adsorption onto eggshell followed the pseudo-second-order kinetic model. As can be observed from Table 1, the *R*^2^ values of the pseudo-second-order kinetic model are higher than those of pseudo-first-order, the value was 1 in all cases analyzed. These results suggested that chemical adsorption was the rate-limiting step that controls this adsorption process. Chemisorption occurs when strong interactions, including hydrogen bonding and covalent and ionic bond formation, occur between the adsorbate and the solid surface. The endpoint for chemisorption is when all the active sites on the solid surface are occupied by chemisorbed molecules. Ehrampoush et al. observed similar kinetics in the adsorption of Reactive Red 123 dye onto eggshell [49], or in the adsorption of Acid Orange 51 onto the ground eggshell powder [16].

The adsorption is a process that follows many steps; firstly, it implicates a transport of dye molecules from the solution to the adsorbent surface, and then a diffusion to the interior of the eggshell could take place [32]. In order to understand the adsorption of DB78 dye onto eggshell, the kinetic of the adsorption process was analyzed using the intraparticle diffusion model, in order to determine if the intraparticle diffusion is the rate-limiting step in the adsorption. This effect was studied by plotting the amount of DB78 dye adsorbed versus the square root of time (Figure 4c).

Figure 4c shows the plot of *q_t_* versus *t*^1/2^ for the intraparticle diffusion of DB78 for the eggshell and different concentrations of dye. Two different straight lines can be distinguished in Figure 4c for the range of concentrations analyzed, indicating that two or more forces are influencing the adsorption process; in this case, chemisorption and intraparticle diffusion played essential roles in the adsorption of DB78 onto eggshell (Figure 4c).

The intraparticle diffusion constant (*k_i_*) values are presented in Table 1. The values of *k_i_* and *C* were calculated from the slope and intercept of plots of *q_t_* versus *t*^1/2^. These *k_i_* values increased with increasing dye concentrations. The *R*^2^ values were very different depending on the concentration, ranging from 0.704 to 0.995. The intraparticle diffusion model was not the rate-controlling step because the results did not pass through the origin. When this plot gives rise to a straight line, the adsorption process is controlled by intra-particle diffusion only. However, if the data present multi-linear plots, then two or more steps influence the adsorption process, as shown in Figure 4. Similar results were also reported for the adsorption of Acid Red 14 and Acid Blue 92 onto the microporous and mesoporous eggshell membrane [37].

Conversely, the intercept of each curve is proportional to the boundary layer thickness; a higher intercept indicates a higher effect. This value decreased with increasing dye concentrations for the eggshell; therefore, the intraparticle diffusion model was not the sole rate-controlling step for eggshell, confirming our previous suggestions [32].

### 3.5. Adsorption Equilibrium

**Figure 5:**
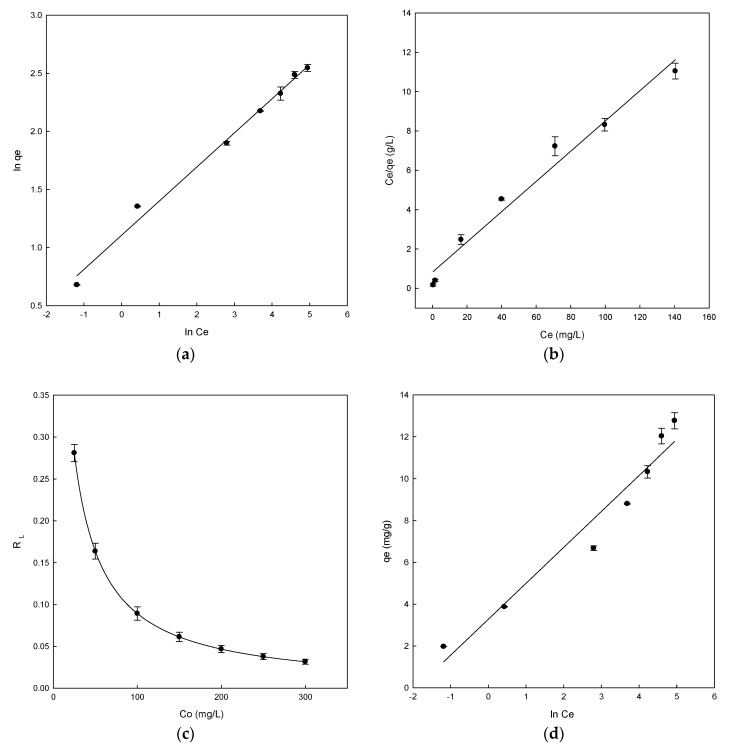
Adsorption isotherms for Direct Blue 78 by eggshell (**a**) Freundlich isotherm, (**b**) Langmuir isotherm, (**c**) separation factor, and (**d**) Temkin isotherm.

The adsorption process of DB78 dye by eggshell adsorbent can be analyzed by fitting the experimental data of adsorption equilibrium to different isotherm models to find the most suitable isotherm to describe the adsorption process. Three well-known models were applied in the present study: Freundlich, Langmuir and Temkin isotherms. The constant isotherm parameters obtained from linear regression describe the equilibrium characteristics of adsorption, and are presented in Table 2. The plot of ln*q_e_* versus ln*C_e_* gave a straight line over the entire concentration range studied in the representation of the Freundlich isotherm, as may be observed in Figure 5a.

Thus, the straight line obtained was used to calculate the parameters *K_F_*, *n_F_*, and *R*^2^. In this case, the *K_F_* was 3.02 and *n_F_* was 3.4 (Table 2). The process is favourable when the *n_F_* value was found in the range between 1 and 10, which was confirmed for eggshell. A good linear determination coefficient (*R*^2^ = 0.991) (Table 2) was obtained. The Freundlich model was the most suitable to describe the adsorption process owing to the high determination coefficient obtained. The linear form of the Langmuir isotherm was obtained by plotting *C_e_*/*q_e_* versus *C_e_*, giving a straight line (Figure 5b). The Langmuir isotherm constants *a_L_/K_L_* and 1/*K_L_* were determined from the slope and intercept of plot (*C_e_/q_e_* vs. *C_e_*), respectively. *K_L_*/*a_L_* is *q_max_* parameter, which is the maximum adsorption capacity of the adsorbent (mg/g) (Figure 5b). The parameters and constants obtained for Langmuir can be observed in Table 2. The maximum adsorption capacity of eggshell was 13 mg/g. Moreover, the value of *R*^2^ was 0.975, but this value was lower than the Freundlich isotherm value.

This *q_max_* value was similar to that of other adsorbents, for example, using chitosan in the adsorption of DB78 [15]; in the removal of various dyes using cellulose by-products, banana, and orange peels [50]; or in the adsorption of methylene blue dye using raw olive stone [20].

According to the results obtained, the Freundlich model fitted better than the Langmuir model. This may demonstrate the presence of heterogeneous adsorption sites on the eggshell surfaces. Similar results were analyzed for the adsorption of Reactive Red 123 dye using eggshell, in which the Freundlich and Langmuir isotherm models provided excellent fit with the highest *R*^2^ value [49]. In another study, Pramanpol and Nitayapat carried out the adsorption of Reactive Yellow 205 dye using various components of eggshells and found a good concordance with the Freundlich model [51]. 

In the Langmuir isotherm, the value of *R_L_* determines if the adsorption process is favourable or unfavourable. A value of *R_L_* in the range between 0 and 1 indicates that the process is favourable. The values obtained for eggshell ranged from 0.281 to 0.032, confirming the adsorption process was favourable (Figure 5c). At low concentrations, the highest *R_L_* values are also observed, indicating that the adsorption is more favourable at those concentrations.

The plot of *q_e_* versus ln *C_e_* shows the representation of the Temkin isotherm (Figure 5d). The Temkin constants *b_T_* and *a_T_* were determined from the slope and intercept, respectively. The *b_T_* value obtained was 1.41 and the *a_T_* value was 6.73, as shown in Table 2. A positive value of *b_T_* indicates that physical and chemical forces were involved in the adsorption process. The value of *R*^2^ was 0.951. The obtained result is lower than the Freundlich and Langmuir determination coefficients, with the Freundlich model being the best isotherm to explain the experimental results.

### 3.6. Thermodynamic Study

**Figure 6:**
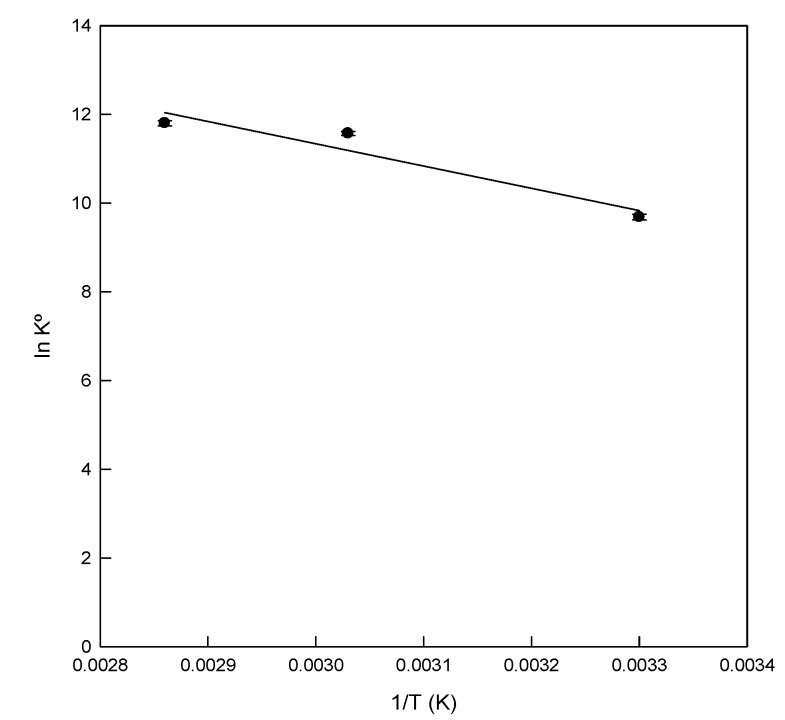
Van’t Hoff plot for the adsorption of Direct Blue 78 onto eggshell at different temperatures.

In order to study the effect of temperature on the adsorption of DB78 by eggshell, the experiments were conducted at three temperatures (Figure 6) at a concentration of 50 mg/L, pH 5, 80 min of contact time, and constant stir (500 rpm). The values of the thermodynamic parameters obtained at different temperatures are presented in Table 3.

The standard free energy (Δ*G*°) of the adsorption of DB78 onto eggshell was −24,417.19 J/mol, −30,886.69 J/mol, and −34376.63 J/mol at temperatures of 29, 55, and 75 °C, respectively. The exergonic values obtained of Δ*G*° indicated that the process is spontaneous at the three temperatures tested, confirming the viability of the process. The enthalpy change (Δ*H*°) was 41,690.71 J/mol. The positive value of Δ*H*° indicated the endothermic nature of the adsorption process. Table 3 shows that the value of Δ*G*° decreased with the increasing temperatures. The value decreased from −24,417.19 to −34,376.63 J/mol, which indicates a clear trend in the process. The adsorption process was favoured at high temperatures.

**Figure 7:**
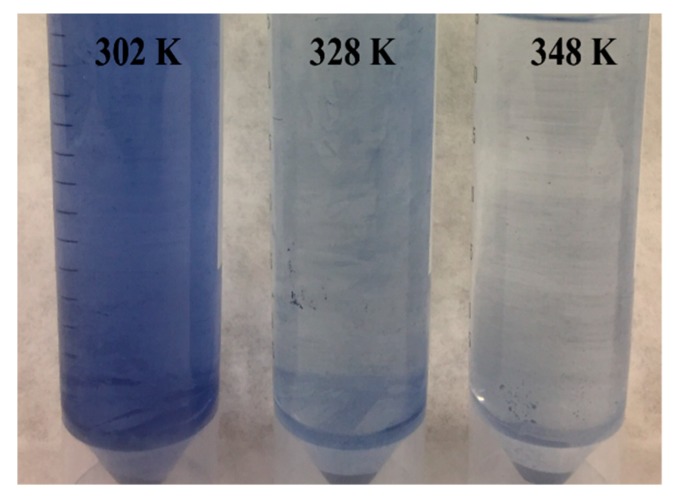
Effect of temperature for the adsorption of Direct Blue 78 onto eggshell at a concentration of 50 mg/L at different temperatures.

As stated before, the thermodynamic analysis revealed that, at 29 °C, there was not an increase in the adsorption abilities of eggshell, however, the best conditions to entrap the dye were achieved at 75 °C. At this temperature, the ability of the adsorbent to adsorb more dye molecules increased, as can be observed in Figure 7.

### 3.7. Desorption and Regeneration of the Adsorbent

Prior to the desorption measurements, it was mandatory to carry out adsorption experiments. After this first adsorption cycle, the adsorbent was put in an alkaline medium (pH 12). In the first desorption cycle, 21% of dye was released from the eggshell; this could explain the electrostatic repulsion between the anionic dye and the negatively charged surface on the adsorbent. The ability of the eggshell to desorb more dye molecules after four consecutive cycles could be attributed to the distribution of charges on the adsorbent surface. After exposing the adsorbent to the same pH solution four times, the adsorbent releases the dye more easily. Arami et al. observed similar results in the adsorption of two different dyes onto the microporous and mesoporous eggshell membrane [37].

**Figure 8:**
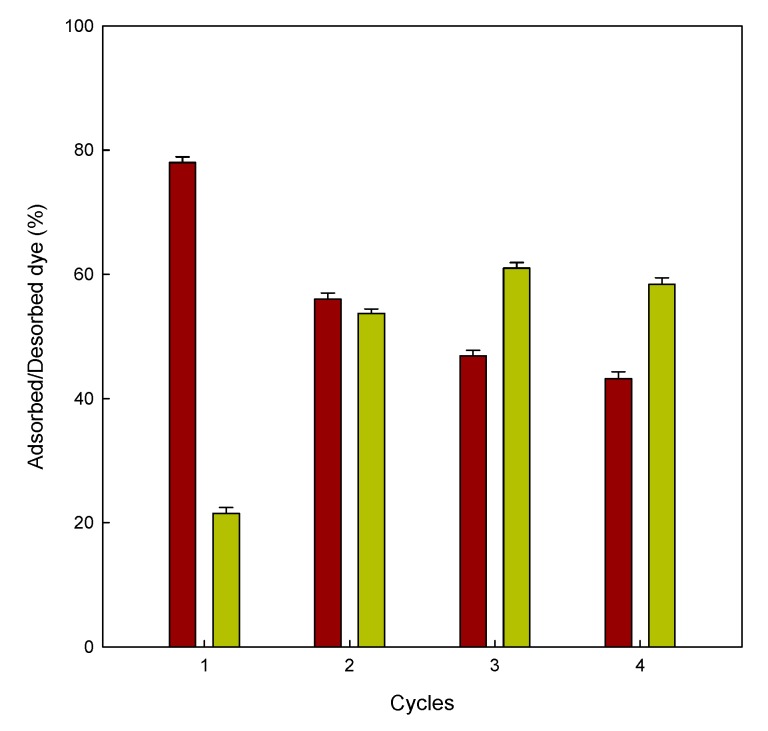
Adsorbed/desorbed dye (%) onto eggshell for four consecutive adsorption (■)/desorption (■) cycles.

This adsorption/desorption cycle was repeated four times (Figure 8); according to the results observed, the adsorption abilities of the eggshell decreased with the increasing the number of cycles. This trend was the opposite for the desorption experiments. The results confirm that the eggshell can be reused in different cycles of adsorption/desorption.

### 3.8. Degradation of DB78 by an H_2_O_2_/Pulsed Light AOP

**Figure 9:**
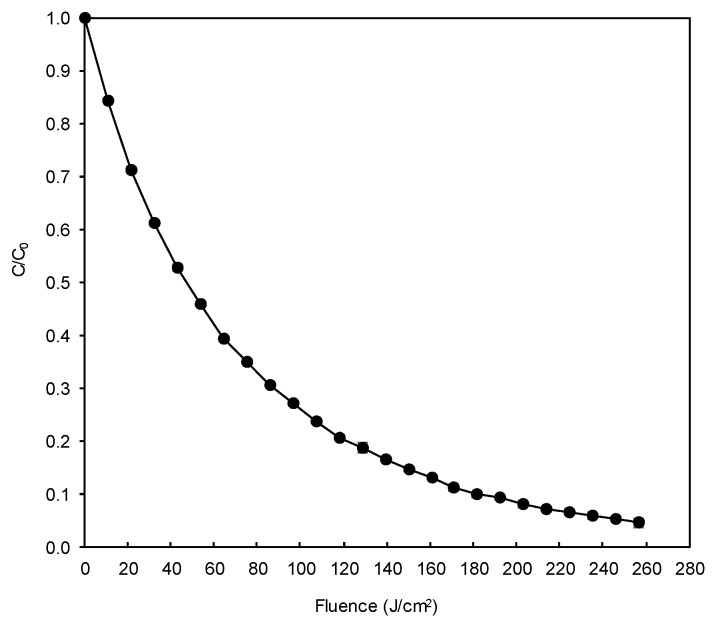
Degradation of DB78 by the H_2_O_2_/pulsed light advanced oxidation process in model wastewater.

The degradation of DB78 by the H_2_O_2_/pulsed light AOP can be observed in Figure 9. The process was able to degrade the dye by more than 95%, which can be considered a significant reduction in the contamination potential of this dye if released to the environment. The respective pseudo-first order degradation rate was 0.012 cm^2^/J (*R*^2^ = 0.9967). Prolonging the treatment would not be efficient as data extrapolation allows predicting 99% degradation after supplying an energy of 383 J/cm^2^, which means expending 49% more energy to increase the degradation by only 4%.

## 4. Conclusions

The present study showed that eggshell can be successfully utilized as a biosorbent for the removal of DB78 dye from water solutions. It was found that the adsorption of DB78 onto eggshell followed pseudo-second-order kinetics. The Freundlich isotherm was the best model to describe adsorption. The maximum adsorption of DB78 onto eggshell was obtained at pH 5 and 0.5 g of adsorbent dosage. According to the Langmuir isotherm, the maximum adsorption capacity of DB78 onto eggshell was 13 mg/g. Taking into account the results obtained for the *n_F_* and *R_L_* parameters, the adsorption is considered as favorable. The analysis of thermodynamic parameters revealed that the adsorption process is endothermic and spontaneous at the three temperatures analyzed. Desorption studies were conducted and the results showed that the eggshell was reusable in different adsorption/desorption cycles. Complementing the adsorption process by an H_2_O_2_/pulsed light advanced oxidation process allows further decreasing the release of pollutant dyes to the environment, proving that the combination of both techniques can be used successfully in the removal of dyes from wastewater at higher concentrations of dye.

## Figures and Tables

**Table 1:** Kinetics parameters of the pseudo-first-order, pseudo-second-order, and intraparticle diffusion models for the adsorption of Direct Blue 78 onto eggshell.

**PFOM ^1^**			**Eggshell**	
***C*_0_ (mg/L)**	***q*_eexp_ (mg/g)**	***q*_ecal_ (mg/g)**	***k*_1_ (min^−1^)**	***R*^2^**
25	1.969	0.773	0.036	0.813
50	3.875	2.905	0.028	0.800
100	6.618	6.742	0.032	0.995
150	8.804	8.652	0.025	0.995
200	10.329	12.838	0.030	0.938
250	11.979	14.125	0.027	0.938
300	12.766	13.658	0.024	0.968
**PSOM ^2^**				
***C*_0_ (mg/L)**	***q*_eexp_ (mg/g)**	***q*_ecal_ (mg/g)**	***k*_2_ (g/mg min)**	***R*^2^**
25	1.969	1.969	0.257	1
50	3.875	3.874	0.066	1
100	6.618	6.618	0.022	1
150	8.804	8.803	0.0129	1
200	10.329	10.331	0.0093	1
250	11.979	11.979	0.0069	1
300	12.766	12.771	0.0061	1
**IDM ^3^**				
***C*_0_ (mg/L)**	***q*_eexp_ (mg/g)**	**(C) (mg/g)**	***k_i_* (mg/g min^1/2^)**	***R*^2^**
25	--	1.414	0.053	0.704
50	--	1.722	0.190	0.778
100	--	1.618	0.462	0.869
150	--	1.111	0.682	0.961
200	--	0.424	0.888	0.951
250	--	0.651	0.999	0.974
300	--	0.896	1.026	0.995

**^1^** PFOM: pseudo-first-order model; **^2^** PSOM: pseudo-second-order model; **^3^** IDM: intraparticle diffusion model.

**Table 2:** Adsorption isotherm constants obtained for eggshell adsorbent.

Isotherm	Parameter	Eggshell
Freundlich	*K_F_* (mg/g) (L/mg)^1/n^	3.02
	*n_F_*	3.40
	*R* ^2^	0.991
Langmuir	*q*_max_ (mg/g)	13.00
	*K_L_* (L/g)	1.20
	*a_L_* (L/mg)	0.093
	*R* ^2^	0.975
	*R_L_*	0.281–0.032
Temkin	*a_T_* (L/g)	6.73
	*b_T_* (J/mol)	1.41
	*R* ^2^	0.951

**Table 3:** Thermodynamic parameters for the adsorption of Direct Blue 78 onto eggshell at different temperatures.

**Eggshell**	***T* (K)**	**Δ*G*° (J/** **mol)**	**Δ*H*° (J/** **mol** **)**	**Δ*S*° (J/** **mol** **)**
	302	−24,417.19	41,690.71	219.24
	328	−30,886.69	-	-
	348	−34,376.63	-	-
